# Non-contiguous finished genome sequence and description of *Bacillus massilioalgeriensis* sp. nov.

**DOI:** 10.4056/sigs.5459590

**Published:** 2014-03-30

**Authors:** Esma Bendjama, Lotfi Loucif, Seydina M. Diene, Caroline Michelle, Djamila Gacemi-Kirane, Jean-Marc Rolain

**Affiliations:** 1 Unité de recherche sur les maladies infectieuses et tropicales émergentes (URMITE), UMR CNRS, IHU Méditerranée Infection, Faculté de Médecine et de Pharmacie, Aix-Marseille-Université, Marseille, France.; 2 Département de Biochimie, Faculté des Sciences, Université Badji Mokhtar, Annaba, Algérie; 3 Département des Sciences Biologiques, Faculté des Sciences, Université El Hadj Lakhdar, Batna, Algérie.

**Keywords:** *Bacillus massilioalgeriensis*, hypersaline environments, sediments, genome, taxono-genomics

## Abstract

Strain EB01^T^ sp. nov. is the type strain of *Bacillus massilioalgeriensis*, a new species within the genus *Bacillus*. This strain, whose genome is described here, was isolated from sediment sample of the hypersaline lake Ezzemoul sabkha in northeastern Algeria. *B. massilioalgeriensis* is a facultative anaerobic Gram-positive bacillus. Here we describe the features of this organism, together with the complete genome sequence and annotation. The 5,269,577 bp long genome contains 5,098 protein-coding and 95 RNA genes, including 12 rRNA genes.

## Introduction

*Bacillus massilioalgeriensis* sp. nov. strain EB01^T^ (= CSUR P857 = DSM 27334) is the type strain of *B. massilioalgeriensis* sp. nov. It is a new Gram-positive, facultatively anaerobic, motile, indole-negative, rod shaped bacterium with rounded ends. It was isolated from a sediment sample from the hypersaline lake Ezzemoul sabkha in the Oum-El-Bouaghi region in northeastern Algeria, which is an important wintering and resting site for several species of waterbirds, including the Greater Flamingo. This site is one of the Ramsar convention wetlands (http://www.ramsar.org). The genus *Bacillus* was created by Cohn about 142 years ago [1],and mainly comprises Gram-positive, rod-shaped, aerobic or facultatively anaerobic, spore-forming bacteria. The genus includes 279 species and 7 subspecies with validly published names [2]. Members of *Bacillus* genus are ubiquitous in nature, ranging from freshwater to marine sediments and from hot springs and desert sands to Arctic soils; many strains have been isolated from the gastrointestinal tracts of various insects and animals, from vegetation and from food [3]. *Bacillus* strains are biotechnologically priceless because of their high capacity to produce a wide range of antimicrobial compounds, enzymes and other metabolites that can be used in industry [4,5]. Some species of *Bacillus* are pathogenic, such as *B. anthracis* (responsible for causing anthrax) [6] and *B. cereus* (a major cause of food poisoning) [7]. Others are opportunists in immunocompromised patients, and may also be involved in various human infections, including pneumonia, endocarditis, ocular, cutaneous, bone or central nervous system infections and bacteremia [8].The current bacterial taxonomy is based on a combination of various phenotypic and genetic criteria [9,10]. However, the three essential genetic criteria that are used, comprising 16S rRNA gene based phylogeny [11], G+C content, and DNA-DNA hybridization [10,12] exhibit several drawbacks. As a result of the recent decrease in the cost of genomic sequencing, it has been proposed that whole genome sequencing information and MALDI-TOF spectrum [13] be combined with the main phenotypic characteristics as a polyphasic approach strategy (taxono-genomics) to describe new bacterial taxa [14-26].

Here we present a summary classification and a set of features for *B. massilioalgeriensis* sp. nov. strain EB01^T^ together with the description of the complete genome sequence and annotation. These characteristics support the circumscription of the species *B. massilioalgeriensis*.

## Classification and features

**Figure 1:**
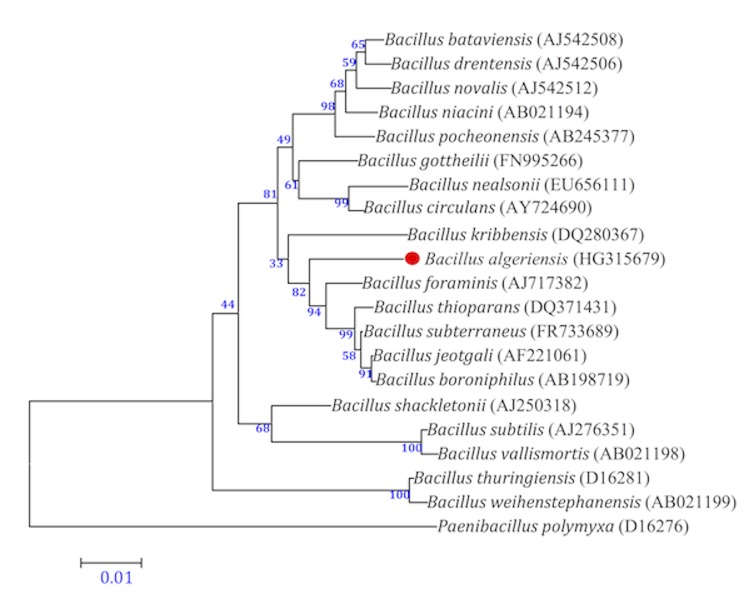
A consensus phylogenetic tree based on 16S rRNA gene sequence comparisons, highlighting the position of strain EB01^T^ *Bacillus massilioalgeriensis* relative to other type strains within the *Bacillus* genus. GenBank accession numbers are displayed in parentheses. Sequences were aligned using CLUSTALW, and phylogenetic inferences made using the neighbor-joining method [38] within the MEGA 5 software [39]. Numbers above the nodes are percentages of bootstrap values from 1,000 replicates that support the node. *Paenibacillus polymyxa* was used as the outgroup. The scale bar represents 0.01 substitutions per nucleotide position.

In July 2012, a sediment sample was aseptically collected in sterile bottles, 15 cm below the evaporite crust of the hypersaline lake Ezzemoul sabkha of Oum-El-Bouaghi region in northeastern Algeria. Samples were transferred in a cooler (4°C) to our lab in Algeria. Samples were processed the same day. Sediments were diluted 1:10 v/v with sterile saline water (0.9% NaCl) and vigorously shaken, Tenfold serial dilutions (10^-1^-10^-5^) of the sediment suspension were plated in Nutrient Agar (NA) medium (meat extract 1 g/l, peptone 5 g/l, yeast extract 2 g/l, sodium chloride 5 g/l, agar 15 g/l) and the plates were incubated at 30°C for 24-72 h. In order to obtain a pure culture, colonies were transferred to fresh NA medium. *Bacillus massilioalgeriensis* sp. nov. strain EB01^T^ (Table 1) was isolated in July 2012 by cultivation under aerobic conditions at 30°C. This strain exhibited a 97.0% 16S rRNA nucleotide sequence similarity with *Bacillus subterraneus* type strain DSM13966^T^ (Figure 1), the phylogenetically closest validly published *Bacillus* species. These values were lower than the 98.7% 16S rRNA gene sequence threshold recommended by Stackebrandt and Ebers to delineate a new species without carrying DNA DNA hybridizidation [11].

**Table 1:** Classification and general features of *Bacillus massilioalgeriensis* strain EB01^T^

**MIGS ID**	**Property**	**Term**	**Evidence code^a^**
		Domain *Bacteria*	TAS [27]
		Phylum *Firmicutes*	TAS [28-30]
		Class *Bacilli*	TAS [31,32]
	Current classification	Order *Bacillales*	TAS [33,34]
		Family *Bacillaceae*	TAS [33,35]
		Genus *Bacillus*	TAS [1,33,36]
		Species *Bacillus massilioalgeriensis*	IDA
		Type strain EB01^T^	IDA
	Gram stain	Positive	IDA
	Cell shape	Rod-shaped	IDA
	Motility	Motile	IDA
	Sporulation	Sporulating	IDA
	Temperature range	Between 37°C and 55°C	IDA
	Optimum temperature	37°C	IDA
MIGS-6.3	Salinity	Growth in LB medium + 0-2.5% NaCl	IDA
MIGS-22	Oxygen requirement	Facultative anaerobic	IDA
	Carbon source	Unknown	NAS
	Energy source	Unknown	NAS
MIGS-6	Habitat	Hypersaline sediment sample	IDA
MIGS-15	Biotic relationship	Free living	IDA
MIGS-14	Pathogenicity Biosafety level Isolation	Unknown 2 Sediment of Ezzemoul Sabkha Lake	NAS NAS IDA
MIGS-4	Geographic location	Algeria	IDA
MIGS-5	Sample collection time	July 2012	IDA
MIGS-4.1	Latitude	35.88167	IDA
MIGS-4.1	Longitude	6.503272	IDA
MIGS-4.3	Depth	Unknown	NAS
MIGS-4.4	Altitude	800 m	IDA

^a^Evidence codes - IDA: Inferred from Direct Assay, TAS: Traceable Author Statement (i.e., a direct report exists in the literature), NAS: Non traceable Author Statement (i.e., not directly observed for the living, isolated sample, but based on a generally accepted property for the species, or anecdotal evidence). These evidence codes are from the Gene Ontology project [37]. If the evidence is IDA, then the property was directly observed for a live isolate by one of the authors or an expert mentioned in the acknowledgements.

**Figure 2:**
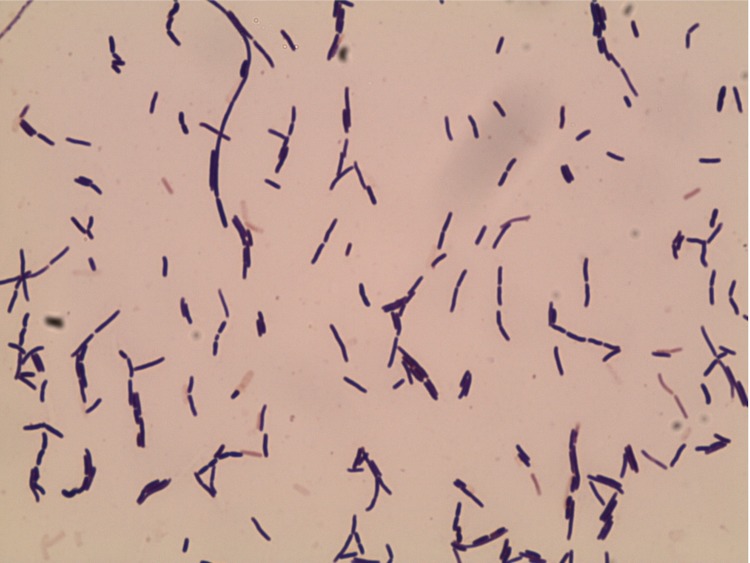
Gram stain of *B. massilioalgeriensis* strain EB01^T^.

**Figure 3:**
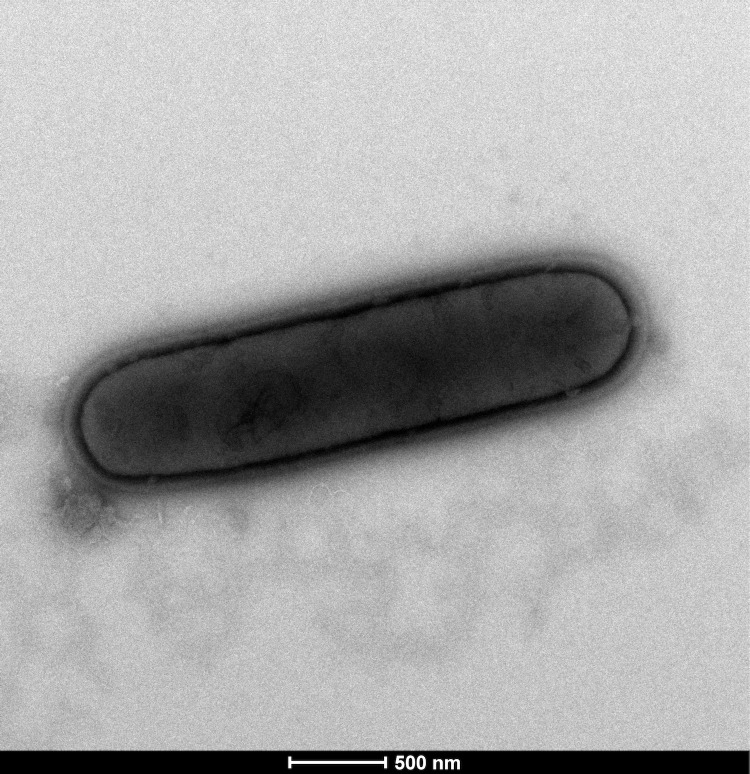
Transmission electron micrograph of *B. massilioalgeriensis* strain EB01^T^ made using a Technai G^2^ Cryo (FEI) at an operating voltage of 200 kV. The scale bar represents 500 nm.

Six different growth temperatures (25, 30, 37, 45, 50 and 55°C), nine NaCl concentrations (0, 2.5, 5, 7.5, 10, 15, 20, 25, 30%) and ten pHs (5, 6, 6.5, 7, 7.5, 8, 8.5, 9, 10, 11) were tested. Growth occurred at all tested temperatures, however the optimal growth was observed at 37°C, between 0% and 2.5% NaCl concentration and pH in the range of 6.5-9 (optimum at pH 7). Colony morphology was observed on sheep blood agar (BioMerieux) after 24 h of aerobic incubation under optimal growth conditions, the colonies of strain EB01^T^ were circular, light yellow, smooth and 2 mm in diameter. Growth of the strain was tested in anaerobic and microaerophilic atmospheres using GasPak EZ Anaerobe Pouch (Becton, Dickinson and Company) and CampyGen Compact (Oxoid) systems, respectively, and in aerobic atmosphere, with or without 5% CO_2_. Growth was achieved under aerobic (with and without CO2) and microaerophilic conditions but weak growth was observed under anaerobic conditions. Gram staining showed Gram-positive rods (Figure 2). Cells grown on agar sporulate. A motility test was positive. The size of cells were determined by negative staining transmission electron microscopy on a Technai G^2^ Cryo (FEI) at an operating voltage of 200 kV, the rods have a length ranging from 2.4 μm to 4.9 μm (mean 3.6 μm) and a diameter ranging from 0.7 μm to 1.1 μm (mean 0.8 μm) (Figure 3).

Strain EB01^T^ exhibited catalase activity but oxidase activity was negative. Using the commercially available API 50CH system (BioMerieux) according to the manufacturer’s instructions, a weak positive reaction was observed for D-ribose, D-glucose, D-fructose, methyl α-D-glucopyranoside, N-acetylglucosamine, D-maltose, D-lactose, D-melibiose, D-saccharose, D-trehalose, D-tagatose, and hydrolysis of starch. Other tests were negative. Using the API ZYM system (BioMerieux), positive reactions were observed for alkaline phosphatase, esterase (C4), esterase lipase (C8), leucine arylamidase, α-chymotrypsin, β-glucuronidase, α-glucosidase, N-acetyl-glucosaminidase and a weak positive reaction was observed for acid phosphatase. The nitrate reduction and β-galactosidase reaction was also positive, but urease and indole production were negative. *B. massilioalgeriensis* was susceptible to amoxicillin, nitrofurantoin, erythromycin, doxycycline, rifampicin, vancomycin, gentamicin, imipenem, trimethoprim-sulfamethoxazole, ciprofloxacin, ceftriaxone and amoxicillin-clavulanic acid, but resistant to nalidixic acid.

When compared to other *Bacillus* species [40-48], *Bacillus massilioalgeriensis* sp. nov. strain EB01^T^ exhibited the phenotypic differences detailed in (Table 2).

**Table 2:** Differential phenotypic characteristics between *B. massilioalgeriensis* sp. nov. strain EB01^T^ and phylogenetically close *Bacillus* species^†^.

**Characteristic**	*B. ma*	*B. su*	*B. fo*	*B. je*	*B. th*	*B. bo*	*B. ba*	*B. ne*	*B. kr*
Cell-diameter(µm)	0.7-1.1	0.5-0.8	1	0.8-1.1	0.5-0.7	0.5-0.9	0.7-1.2	1	1.4-2.0
Oxygen requirement	facultative anaerobic	facultative anaerobic	aerobic	facultative anaerobic	aerobic	na	facultative anaerobic	facultative anaerobic	aerobic
Gram strain	+	-	+	V	V	+	+ or V	+	+
NaCl range (%,w/v)	0-2.5	0-9	0-3	0-13	0-5	0-7	na	0-8	0-6
Motility	+	+	na	+	+	+	+	+	+
Endospore formation	+	-	-	+	+	+	+	+	+
**Production of**									
Alkaline phosphatase	+	na	na	na	na	+	na	na	na
Acid phosphatase	w	na	na	na	na	na	na	na	na
Catalase	+	+	+	+	+	+	na	+	+
Oxidase	-	-	+	-	-	+	na	na	-
Nitrate reductase	+	+	+	+	+	-	+	-	-
Urease	-	-	+	+	-	-	-	-	-
α-galactosidase	-	na	na	na	na	na	na	na	na
β-galactosidase	-	-	+	na	na	-	na	+	na
β-glucuronidase	+	na	na	na	na	na	na	na	+
N-acetyl-β-glucosaminidase	+	na	na	na	na	na	na	na	na
Indole	-	-	na	-	-	-	-	-	na
Esterase	+	na	na	na	na	na	na	na	+
Esterase lipase	+	na	na	na	na	w	na	na	+
Naphthyl-AS-BI-Phosphohydrolase	-	na	na	na	na	na	na	na	+
Leucine arylamidase	+	na	na	na	na	+	na	na	na
Cystine arylamidase	-	na	na	na	na	w	na	na	na
Valine arylamidase	-	na	na	na	na	w	na	na	+
**Utilization of**									
D-mannose	-	-	+	-	-	+	+	+	-
Amygdalin	-	na	+	-	-	+	w	-	na
L-Arabinose	-	-	+	-	na	na	-	+	-
Cellobiose	-	-	+	+	na	+	+	+	+
Lactose	w	-	+	-	na	na	+	+	+
D-xylose	-	+	+	-	na	na	-	+	+
D-Glucose	w	+	+	+	+	na	+	+	+
Mannitol	-	-	+	-	-	na	+	+	na
Arabinose	-	-	+	-	-	na	-	-	na
L-Xylose	-	+	+	-	na	na	-	na	+
Glycerol	-	+	+	-	na	+	w	+	-
D-Galactose	-	-	+	-	na	na	+	+	na
**Hydrolysis of**									
Starch	w	+	+	+	na	na	v	+	-
Gelatin	-	+	+	+	w	-	+	-	+
**G+C content (mol%)**	42,22	43±1	43,1	41	43,8	41,1-42,2	39,6	na	43,3
**Habitat**	hyersaline sediment	deep subterranean thermal waters	alkaline ground water	fermented seafood	wastewater treatment culture system	soil	soil	spacecraft-assembly facility	soil

^†^(*Bacillus subterraneus* strain COOI3B^T^, *Bacillus foraminis* strain CV53^T^, *Bacillus jeotgali* strain YKJ-10^T^, *Bacillus thioparans* strain BMP-1^T^, *Bacillus boroniphilus* strain T-15Z^T^, *Bacillus bataviensis* strain IDA1115^T^, *Bacillus nealsonii* strain FO-92^T^, *Bacillus kribbensis* strain BT080^T^).

*B. massilioalgeriensis* (*B. ma*), *B. subterraneus* (*B. su*), *B. foraminis* (*B. fo*), *B. jeotgali* (*B. je*), *B. thioparans* (*B. th*), *B. boroniphilus* (*B. bo*), *B. bataviensis* (*B. ba*), *B. nealsonii* (*B. ne*) and *B. kribbensis* (*B. kr*).

+: positive result, -: negative result, var: variable, w: weak positive result, na: data not available.

**Figure 4:**
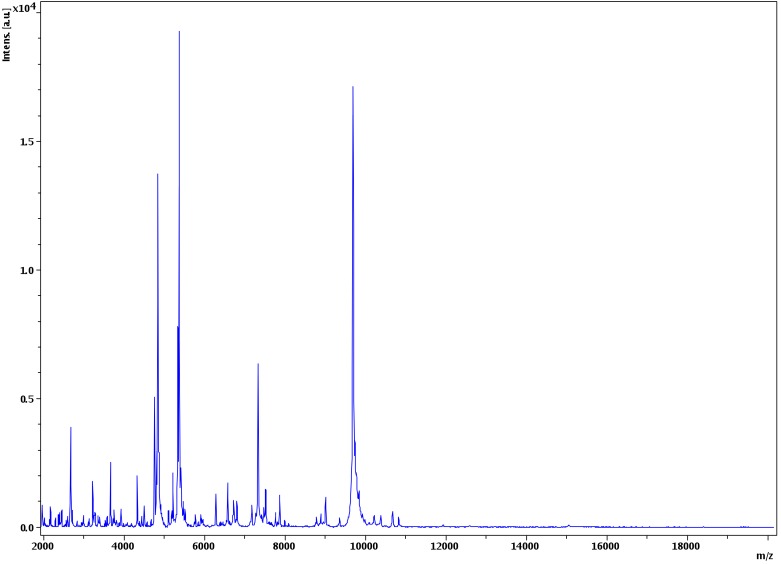
Reference mass spectrum from *B. massilioalgeriensis* strain EB01^T^. Spectra from 12 individual colonies were compared and a reference spectrum was generated.

**Figure 5:**
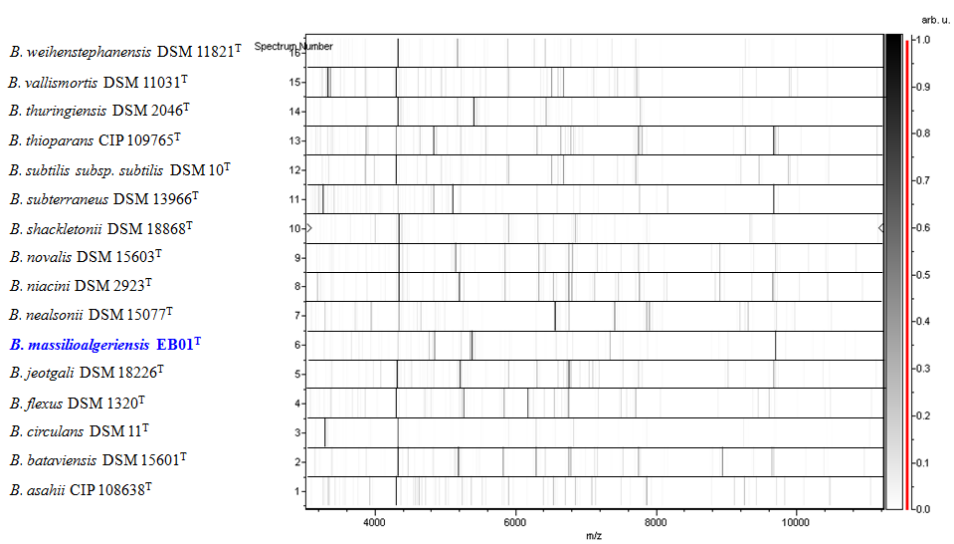
Gel view comparing *Bacillus massilioalgeriensis* EB01^T^ spectra with other members of the *Bacillus* genus (*B. weihenstephanensis, B. vallismortis, B. thuringiensis, B. thioparans, B. subtilis subsp. subtilis, B. subterraneus, B. shackletonii, B. novalis, B. niacini, B. nealsonii, B. jeotgali, B. flexus, B. circulans, B. bataviensis and B. asahii*). The Gel View displays the raw spectra of all loaded spectrum files as a pseudo-electrophoretic gel. The x-axis records the m/z value. The left y-axis displays the running spectrum number originating from subsequent spectra loading. The peak intensity is expressed by a grey scale scheme code. The grey scale bar on the right y-axis indicates the relation between the shade of grey a peak is displayed with and the peak intensity in arbitrary units.

Matrix-assisted laser-desorption/ionization time-of-flight (MALDI-TOF) MS protein analysis was performed as previously described [26,49,50]. Briefly, strain EB01^T^ was plated on 5% sheep blood-enriched Columbia agar (BioMerieux) and incubated for 24 h at 37°C. Isolated bacterial colonies were picked, and then deposited as a thin film in 12 replicates on a MALDI-TOF steel target plate (Bruker Daltonics, Bremen, Germany). The plates were allowed to dry at room temperature. Each deposit was overlaid with 1.5 µl of matrix solution containing α-cyano-hydroxycinnamic acid (Sigma, Saint-Quentin Fallavier, France) saturated with 50% acetonitrile, 2.5% trifluoroacetic acid and high-performance liquid chromatography (HPLC)-grade water, and allowed to co-crystallize with the sample. Measurements were conducted using the Microflex LT spectrometer (Bruker Daltonics). Spectra were recorded in the linear positive ion mode over a mass range of 2 to 20 kDa. The acceleration voltage was 20 kV. Spectra were collected as a sum of 240 shots across a spot. The 12 EB01^T^ spectra were imported into the MALDI BioTyper software (version 3.0, Bruker) and analyzed by standard pattern matching (with default parameter settings) against 6,335 bacterial spectra including 210 spectra from 104 *Bacillus* species, used as reference data, in the BioTyper database. A score enabled the identification, or not, from the tested species: a score > 2 with a validated species enabled the identification at the species level, a score > 1.7 but < 2 enabled the identification at the genus level; and a score < 1.7 did not enable any identification. For strain EB01^T^, the scores obtained ranged from 1.15 to 1.60 thus suggesting that our isolate was a new species. We iadded the spectrum from strain EB01^T^ (Figure 4) to our database. Spectrum differences with other of *Bacillus* species are shown in (Figure 5).

## Genome sequencing information

### Genome project history

The organism was selected for sequencing on the basis of its phylogenetic position and 16S rRNA similarity to other members of the genus *Bacillus*, and is part of a study of *Bacillus* genus diversity in hypersaline lakes of Algeria. It was the 398^th^ genome of a *Bacillus* species and the first genome of *Bacillus massilioalgeriensis* sp. nov. The EMBL accession number is ERP003483 and consists of 46 contigs. Table 3 shows the project information and its association with MIGS version 2.0 compliance [51].

**Table 3:** Project information

**MIGS ID**	**Property**	**Term**
MIGS-31	Finishing quality	High-quality draft
MIGS-28	Libraries used	Nextera XT library
MIGS-29	Sequencing platform	Miseq-Illumina
MIGS-31.2	Sequencing coverage	34×
MIGS-30	Assemblers	Velvet
MIGS-32	Gene calling method	Prodigal
	EMBL Date of Release	January 10, 2014
	EMBL ID	ERP003483
MIGS-13	Project relevance	Study of the *Bacillus* genus diversity in hypersaline lakes of northeastern Algeria

### Growth conditions and DNA isolation

*Bacillus massilioalgeriensis* sp. nov strain EB01^T^, was grown aerobically on 5% sheep blood enriched Columbia agar at 37°C. Three petri dishes were spread and resuspended in a 2 ml sterile Eppendorf tube containing 1ml of TE buffer with acid-washed glass beads (diameter ≤106 µm, Sigma, Saint-Quentin Fallavier, France). Three cycles of shaking were performed using a FastPrep BIO 101 apparatus (Qbiogene, Strasbourg, France) for 15 sec at level 6.5 (full speed). Then, the supernatant was placed in a new tube along with one hundred μl of 10% SDS and 50 µl of Proteinase K (Qiagen GmbH, Hilden, Germany) and incubated over night at 56°C. The digested mixture was used to perform DNA extraction using the classical phenol-chloroform method. The quality of the DNA was checked on an agarose gel (0.8%) stained with SYBR safe.

### Genome sequencing

Genomic DNA of *B. massilioalgeriensis* sp. nov. strain EB01^T^ was sequenced on the MiSeq platform (Illumina, Inc, San Diego CA 92121, USA) with a paired end and barcode strategy in order to be mixed with 7 others genomic projects constructed with the Nextera XT library kit (Illumina).

The gDNA was quantified by a Qubit assay with the high sensitivity kit (Life technologies, Carlsbad, CA, USA) to 34.4 ng/µL and dilution was performed to provide 1 ng of each small genome as input. The “tagmentation” step fragmented and tagged the DNA to generate an optimum insert size of 1.6 kb, validated on a high sensitivity labchip Calliper-Perkin Elmer (Caliper Life Sciences, Inc, Massachusetts, USA). Then limited cycle PCR amplification completed the tags adapters and introduced dual-index barcodes. After purification on Ampure beads (Lifetechnolgies, Carlsbad, CA, USA), the libraries were normalized on specific beads according to the Nextera XT protocol (Illumina). Normalized libraries are pooled into a single library for sequencing on the MiSeq. The pooled single strand library was loaded onto the reagent cartridge and then onto the instrument along with the flow cell. Automated cluster generation and paired-end sequencing with dual index reads was performed in a single 39-hour run with a 2×250 bp read length. Within this pooled run, the index representation was determined to 7.1%. Total information of 2.4 G bases was obtained from a 320 K/mm2 density with 94.9% (5,757,000 clusters) of the clusters passing quality control (QC) filters. From the genome sequencing process, the 775,420 produced Illumina reads for *B. massilioalgeriensis* EB01^T^ were filtered according to the read qualities and sizes using the fastq-mcf program (Ea-utils: command-line tools for processing biological sequencing data) [52]. 714,540 filtered read sequences were kept for genome assembly. The Velvet assembler was used with different kmer values (from 51 to 95) and the best assembly result with kmer value (n=91) producing 46 contigs with sizes from 872 bp to 409,112 bp, was retained for genome annotation.

### Genome annotation

Open Reading Frames (ORFs) were predicted using Prodigal [53] with default parameters. The predicted bacterial protein sequences were searched against the Clusters of Orthologous Groups (COG) database and the GenBank database [54] using BLASTP. Ribosomal RNAs were found by using RNAmmer 2.1 server [55,56] and BLASTn against the GenBank database, whereas the tRNAScanSE tool [57] was used to find tRNA genes. Transmembrane helices and lipoprotein signal peptides were predicted using phobius web server [58]. ORFans were identified if their BLASTP *E*-value was lower than 1e-03 for alignment length greater than 80 amino acids. If alignment lengths were smaller than 80 amino acids, we used an *E*-value of 1e-05. Artemis [59] was used for data management and DNA Plotter [60] was used for visualization of genomic features. To estimate the mean level of nucleotide sequence similarity at the genome level between *B. massilioalgeriensis* sp nov. strain EB01^T^ and seven other *Bacillus* species, we use the Average Genomic Identity of Orthologous gene Sequences (AGIOS) in-house software. Briefly, this software combines the Proteinortho software [61] for pairwise comparison and detection of orthologous proteins between genomes, then retrieves the corresponding genes and determines the mean percentage of nucleotide sequence identity among orthologous ORFs using the Needleman-Wunsch global alignment algorithm.

## Genome properties

**Figure 6:**
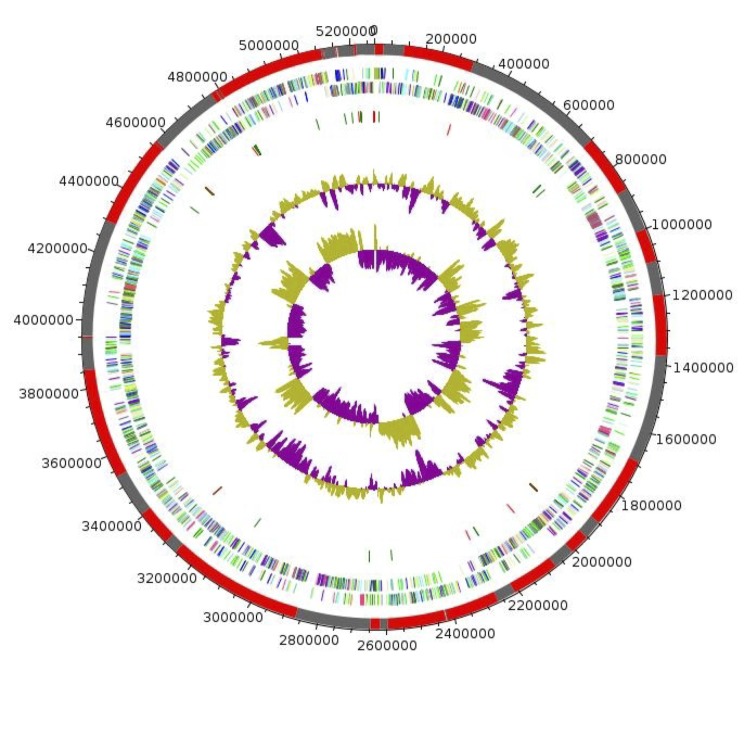
Graphical circular map of the chromosome. From outside to the center: Red and gray bars representing contigs, genes on the forward strand colored by COG categories (only genes assigned to COG), genes on the reverse strand colored by COG categories (only genes assigned to COG), RNA genes (tRNAs green, rRNAs red), GC content. The inner-most circle shows the GC skew, purple and olive indicating negative and positive values, respectively.

The genome is 5,269,577 bp long with 42.22% GC content (Figure 6 and Table 4). It is composed of 46 contigs. Of the 5,193 predicted genes, 5098 were protein-coding genes, and 95 were RNAs (10 genes encode 5S rRNA, 1 gene encodes 16S rRNA, 1 gene encodes 23S rRNA, 83 genes are tRNA genes). A total of 3,217 genes (63.1%) were assigned a putative function (by cogs or by NR blast). 457 genes were identified as ORFans (8.96%). The remaining genes were annotated as hypothetical proteins (1097 genes, 21.52%). The distribution of genes into COGs functional categories is presented in Table 5. The properties and statistics of the genome are summarized in Tables 4 and 5.

**Table 4:** Nucleotide content and gene count levels of the genome

**Attribute**	Value	% of total^a^
Genome size (bp)	5,269,577	100
DNA coding region (bp)	4,342,253	82.4
DNA G+C content (bp)	2,224,760	42.21
Total genes	5,193	100
RNA genes	95	1.82
Protein-coding genes	5,098	98.17
Genes with function prediction	3,217	63.1
Genes assigned to COGs	3,041	59.65
Genes with peptide signals	653	12.8
Genes with transmembrane helices	1,297	25.44

^a^ The total is based on either the size of the genome in base pairs or the total number of protein coding genes in the annotated genome

**Table 5:** Number of genes associated with the 25 general COG functional categories

**Code**	**Value**	**% age**^a^	**Description**
J	176	3.45	Translation, ribosomal structure and biogenesis
A	0	0	RNA processing and modification
K	281	5.51	Transcription
L	165	3.23	Replication, recombination and repair
B	1	0.01	Chromatin structure and dynamics
D	33	0.64	Cell cycle control, mitosis and meiosis
Y	0	0	Nuclear structure
V	63	1.23	Defense mechanisms
T	144	2.82	Signal transduction mechanisms
M	156	3.06	Cell wall/membrane biogenesis
N	48	0.94	Cell motility
Z	0	0	Cytoskeleton
W	0	0	Extracellular structures
U	50	0.98	Intracellular trafficking and secretion
O	110	2.15	Posttranslational modification, protein turnover, chaperones
C	206	4.04	Energy production and conversion
G	277	5.43	Carbohydrate transport and metabolism
E	370	7.25	Amino acid transport and metabolism
F	82	1.60	Nucleotide transport and metabolism
H	109	2.13	Coenzyme transport and metabolism
I	142	2.78	Lipid transport and metabolism
P	213	4.17	Inorganic ion transport and metabolism
Q	86	1.68	Secondary metabolites biosynthesis, transport and catabolism
R	521	10.21	General function prediction only
S	327	6.41	Function unknown
-	2057	40.34	Not in COGs

^a^ The total is based on the total number of protein coding genes in the annotated genome

## Comparison with other species *Bacillus* genomes

Here, we compared the genome of *B. massilioalgeriensis* strain EB01^T^ with those of *B. kribbensis* strain DSM 17871, *B. nealsonii* strain AAU1, *B. bataviensis* strain LMG 21833, *B. subtilis subsp. subtilis* strain 168, *B. vallismortis* strain DV1-F-3, *B. thuringiensis* strain BMB171 and *B. weihenstephanensis* strain KBAB4 (Table 6). The draft genome of *B. massilioalgeriensis* (5.26Mb) is larger in size than those of *B. kribbensis, B. nealsonii, B. subtilis subsp. subtilis* and *B. vallismortis* (5.05, 4.98, 4.22 and 3.88 Mb, respectively) but smaller than those of, *B. bataviensis*, *B. thuringiensis* and *B. weihenstephanensis* (5.37, 5.64 and 5.87 Mb, respectively). *B. massilioalgeriensis* has a lower G+C content than *B. kribbensis, B. subtilis subsp. subtilis* and *B. vallismortis* (42.22% vs 43%, 43.5% and 43.8%, respectively) but higher than *B. nealsonii, B. bataviensis, B. thuringiensis* and *B. weihenstephanensis* (42.22% vs 35.1%, 39.6%, 35.2% and 35.5%, respectively). *B. massilioalgeriensis* has more predicted protein coding genes (5,098) than *B. kribbensis, B. nealsonii, B. subtilis subsp. subtilis* and *B. vallismortis* (4,918, 4,789, 4,175 and 4,097, respectively) but fewer protein coding genes than *B. bataviensis, B. thuringiensis* and *B. weihenstephanensis* (5,207, 5,352 and 5,653, respectively). In addition, *B. massilioalgeriensis* shared 1,804, 1,778, 2,017, 1,768, 1,985, 1,541, 1,863 orthologous genes with *B. thuringiensis, B. nealsonii, B. bataviensis, B. subtilis subsp. subtilis, B. kribbensis, B. vallismortis, B. weihenstephanensis* respectively.

The average nucleotide sequence identity of orthologous genes ranges from 64.54 to 91.06% among the 8 *Bacillus* species, and from 64.77 to 69.33% between *Bacillus massilioalgeriensis* and the other compared genomes (Table 6), thus confirming its new species status.

**Table 6A:** Genomic comparison of *B. massilioalgeriensis* sp. nov. strain EB01^T^ with seven other *Bacillus* species**^†^**.

**Species**	**Strain**	**Genome accession number**	**Genome size (Mb)**	**G+C content**
*Bacillus massilioalgeriensis*	EB01^T^	ERP003483	5.26	42.22
*Bacillus kribbensis*	DSM 17871	AUMQ00000000.1	5.05	43
*Bacillus nealsonii*	AAU1	ASRU00000000.1	4.98	35.1
*Bacillus bataviensis*	LMG 21833	AJLS00000000.1	5.37	39.6
*Bacillus subtilis subsp. subtilis*	168	NC_000964.3	4.22	43.5
*Bacillus vallismortis*	DV1-F-3	AFSH00000000.1	3.88	43.8
*Bacillus thuringiensis*	BMB171	NC_014171.1	5.64	35.2
*Bacillus weihenstephanensis*	KBAB4	NC_010184.1	5.87	35.5

**Table:** **^†^** species and strain names, genome accession numbers, sizes and G+C contents.

**Table 6B:** Genomic comparison of *B. massilioalgeriensis* sp. nov. strain EB01^T^ with seven other *Bacillus* species^†^.

Species	*B. th*	*B.ne*	*B.ba*	*B. subt*	***B. ma***	*B. kr*	*B.va*	*B.we*
*B. thuringiensis*	**5,352**	1,642	1,825	1,786	1,804	1,783	1,548	2,369
*B. nealsonii*	67.32	**4,789**	1,746	1,679	1,778	1,751	1,450	1,702
*B. bataviensis*	66.65	68.78	**5,207**	1,812	2,017	1,997	1,567	1,904
*B.subtilis subsp.subtilis*	65.35	65.74	66.03	**4,175**	1,768	1,841	2,016	1,838
***B. massilioalgeriensis***	64.77	66.61	69.33	65.44	**5,098**	1,985	1,541	1,863
*B. kribbensis*	64.54	66.05	67.20	65.86	66.92	**4,918**	1,604	1,864
*B. vallismortis*	64.56	65.05	65.54	91.06	64.92	65.30	**4,097**	1,592
*B. weihenstephanensis*	89.95	67.27	66.70	65.46	64.87	64.56	64.70	**5,653**

^†^Numbers of orthologous protein shared between genomes (above diagonal), average percentage similarity of nucleotides corresponding to orthologous proteins shared between genomes (below diagonal). Bold numbers indicate numbers of proteins per genome.

*B. thuringiensis* (*B. th*), *B. nealsonii* (*B. ne*), *B. bataviensis* (*B. ba*), *B. subtilis subsp. subtilis* (*B. subt*), *B. massilioalgeriensis* (*B. ma*), *B. kribbensis* (*B. kr*), *B. vallismortis* (*B. va*) and *B. weihenstephanensis* (*B. we*).

## Conclusion

On the basis of phenotypic (Table 2), phylogenetic and genomic analyses (taxonogenomics) (Table 6), we formally propose the creation of *Bacillus massilioalgeriensis* sp. nov. that contains the strain EB01^T^. This strain has been found in hypersaline lacustrine sediment sample collected from Algeria.

### Description of *Bacillus massilioalgeriensis* sp. nov EB01^T^

*Bacillus massilioalgeriensis* (ma.sil.io.al.ge.ri.en’sis. L. gen. masc. n. *massilioalgeriensis*, combination of Algeria, where strain EB01^T^ was isolated and Massilia, the Latin name of Marseille, where the strain was sequenced). Strain EB01^T^ is a facultative anaerobic Gram-positive, endospore-forming, motile and rod shaped bacterium with rounded ends. Growth is achieved aerobically between 30 and 55°C (optimum 37°C), between 0% and 2.5% NaCl concentration and pH in the range of 6.5-9 (optimum at pH 7). Growth is also observed in microaerophilic atmosphere, however, weak growth was observed under anaerobic conditions. After 24h growth on 5% sheep blood-enriched Columbia agar (BioMerieux) at 37°C, bacterial colonies were smooth, light yellow with 2 mm in diameter. Cells have a length ranging from 2.4 μm to 4.9 μm (mean 3.6 μm) and a diameter ranging from 0.7 μm to 1.1 μm (mean 0.8 μm).

Catalase positive but oxidase negative. Using the commercially available API 50CH system (BioMerieux) according to the manufacturer’s instructions, a weak positive reaction was observed for D-ribose, D-glucose, D-fructose, methyl α-D-glucopyranoside, N-acetylglucosamine, D-maltose, D-lactose, D-melibiose, D-saccharose, D-trehalose, D-tagatose, and hydrolysis of starch. Other tests were negative. Using the API ZYM system (BioMerieux), positive reactions were observed for alkaline phosphatase, esterase (C4), esterase lipase (C8), leucine arylamidase, α chymotrypsin, β-glucuronidase, α-glucosidase, N-acetyl-glucosaminidase and a weak positive reaction was observed for acid phosphatase. The nitrate reduction and β-galactosidase reaction was also positive, but urease and indole production were negative. *B. massilioalgeriensis* was susceptible to amoxicillin, nitrofurantoin, erythromycin, doxycycline, rifampin, vancomycin, gentamycin, imipenem, trimethoprim-sulfamethoxazole, ciprofloxacin, ceftriaxone and amoxicillin/clavulanic acid, but resistant to nalidixic acid.

The G+C content of the genome is 42.22. The 16S rRNA and genome sequences are deposited in GenBank under accession numbers HG315679 and EMBL database under accession number ERP003483, respectively. The type strain EB01^T^ (= CSUR P857 = DSM 27334) was isolated from sediment sample of the hypersaline lake Ezzemoul sabkha of Oum-El-Bouaghi region in northeastern Algeria.

## References

[1] Cohn F Untersuchungen über Bakterien. Beitr Biol Pflanz 1872; 1:127-224

[2] Parte AC LPSN-list of prokaryotic names with standing in nomenclature. Nucleic Acids Res 2013; 42:613-616 10.1093/nar/gkt1111 24243842 PMC3965054

[3] Nicholson WL Roles of *Bacillus* endospores in the environment. Cell Mol Life Sci 2002; 59:410-416 10.1007/s00018-002-8433-7 11964119 PMC11337551

[4] Moshafi MH Forootanfar H Ameri A Shakibaie M Noudeh G Razavi M Antimicrobial activity of *Bacillus* sp. strain FAS1 isolated from soil. Pak J Pharm Sci 2011; 24:269-275 21715259

[5] Bumpus SB Evans BS Thomas PM Ntai I Kelleher NL A proteomics approach to discovering natural products and their biosynthetic pathways. Nat Biotechnol 2009; 27:951-960 10.1038/nbt.1565 19767731 PMC2782881

[6] Jernigan JA Stephens DS Ashford DA Omenaca C Topiel MS Galbraith M Tapper M Fisk TL Zaki S Popovic T Bioterrorism-related inhalational anthrax: the first 10 cases reported in the United States. Emerg Infect Dis 2001; 7:933-944 10.3201/eid0706.010604 11747719 PMC2631903

[7] Bottone EJ *Bacillus cereus*, a volatile human pathogen. Clin Microbiol Rev 2010; 23:382-398 10.1128/CMR.00073-09 20375358 PMC2863360

[8] Mandell GL, Bennett JE, Dolin R. Principles and Practice of Infectious Diseases. Elsevier **2010**, 4320p.

[9] Stackebrandt E Frederiksen W Garrity GM Grimont PA Kämpfer P Maiden MC Nesme X Rosselló-Mora R Swings J Trüper HG Report of the ad hoc committee for the re-evaluation of the species definition in bacteriology. Int J Syst Evol Microbiol 2002; 52:1043-1047 10.1099/ijs.0.02360-0 12054223

[10] Tindall BJ Rossello-Mora R Busse HJ Ludwig W Kampfer P Notes on the characterization of prokaryote strains for taxonomic purposes. Int J Syst Evol Microbiol 2010; 60:249-266 10.1099/ijs.0.016949-0 19700448

[11] Stackebrandt E Ebers J Taxonomic parameters revisited: tarnished gold standards. Microbial To-day 2006; 33:152-155

[12] Rossello-Mora R. DNA-DNA Reassociation Methods Applied to Microbial Taxonomy and Their Critical Evaluation. In: Stackebrandt E (ed), Molecular Identification, Systematics, and population Structure of Prokaryotes. Springer, Berlin **2006** p. 23-50.

[13] Welker M Moore ER Applications of whole-cell matrix-assisted laser-desorption/ionization time-of-flight mass spectrometry in systematic microbiology. Syst Appl Microbiol 2011; 34:2-11 10.1016/j.syapm.2010.11.013 21288677

[14] Ramasamy D, Mishra AK, Lagier JC, Padhmanabhan R, Rossi M,Sentausa E, Raoult D, Fournier PE. A polyphasic strategy incorporating genomic data for the taxonomic description of novel bacterial species. *Int J Syst Evol Microbiol* 2014; **64**:384-391. 10.1099/ijs.0.057091-0 24505076

[15] Ramasamy D Lagier JC Gorlas A Raoult D Fournier PE Non contiguous-finished genome sequence and description of *Bacillus massiliosenegalensis* sp. nov. Stand Genomic Sci 2013; 8:264-278 10.4056/sigs.3496989 23991258 PMC3746431

[16] Keita MB Diene SM Robert C Raoult D Fournier PE Bittar F Non-contiguous finished genome sequence and description of *Bacillus massiliogorillae* sp. nov. Stand Genomic Sci 2013; 9:93-105 10.4056/sigs.4388124 24501648 PMC3910557

[17] Mishra AK Pfleiderer A Lagier JC Robert C Raoult D Fournier PE Non-contiguous finished genome sequence and description of *Bacillus massilioanorexius* sp. nov. Stand Genomic Sci 2013; 8:465-479 10.4056/sigs.4087826 24501631 PMC3910694

[18] Kokcha S Mishra AK Lagier JC Million M Leroy Q Raoult D Fournier PE Non contiguous-finished genome sequence and description of *Bacillus timonensis* sp. nov. Stand Genomic Sci 2012; 6:346-355 10.4056/sigs.2776064 23408487 PMC3558959

[19] Mishra AK Lagier JC Rivet O Raoult D Fournier PE Non-contiguous finished genome sequence and description of *Paenibacillus senegalensis* sp. nov. Stand Genomic Sci 2012; 7:70-81 10.4056/sigs.3056450 23459006 PMC3577113

[20] Mishra AK Lagier JC Robert C Raoult D Fournier PE Genome sequence and description of *Timonella senegalensis* gen. nov., sp. nov., a new member of the suborder *Micrococcinae.* Stand Genomic Sci 2013; 8:318-335 10.4056/sigs.3476977 23991262 PMC3746429

[21] Ramasamy D Lagier JC Nguyen TT Raoult D Fournier PE Non contiguous-finished genome sequence and description of of *Dielma fastidiosa* gen. nov., sp. nov., a new member of the Family *Erysipelotrichaceae*. Stand Genomic Sci 2013; 8:336-351 10.4056/sigs.3567059 23991263 PMC3746426

[22] Mishra AK Hugon P Lagier JC Nguyen TT Couderc C Raoult D Fournier PE Non contiguous-finished genome sequence and description of *Enorma massiliensis* gen. nov., sp. nov., a new member of the Family *Coriobacteriaceae.* Stand Genomic Sci 2013; 8:290-305 10.4056/sigs.3426906 23991260 PMC3746427

[23] Hugon P Mishra AK Lagier JC Nguyen TT Couderc C Raoult D Fournier PE Non contiguous-finished genome sequence and description of *Brevibacillus massiliensis* sp. nov. Stand Genomic Sci 2013; 8:1-14 10.4056/sigs.3466975 23961307 PMC3739172

[24] Lagier JC El Karkouri K Mishra AK Robert C Raoult D Fournier PE Non-contiguous finished genome sequence and description of *Enterobacter massiliensis* sp. nov. Stand Genomic Sci 2013; 7:399-412 10.4056/sigs.3396830 24019988 PMC3764934

[25] Mishra AK Lagier JC Nguyen TT Raoult D Fournier PE Non-contiguous finished genome sequence and description of *Peptoniphilus senegalensis* sp. nov. Stand Genomic Sci 2013; 7:370-381 10.4056/sigs.3366764 24019986 PMC3764932

[26] Roux V ElKarkouri K Lagier JC Robert C Raoult D Non-contiguous finished genome sequence and description of *Kurthia massiliensis* sp. nov. Stand Genomic Sci 2012; 7:221-232 10.4056/sigs.3206554 23407462 PMC3569394

[27] Woese CR Kandler O Wheelis ML Towards a natural system of organisms: proposal for the domains *Archaea*, *Bacteria*, and *Eucarya*. Proc Natl Acad Sci USA 1990; 87:4576-4579 10.1073/pnas.87.12.4576 2112744 PMC54159

[28] Gibbons NE Murray RGE Proposals concerning the higher taxa of bacteria. Int J Syst Bacteriol 1978; 28:1-6 10.1099/00207713-28-1-1

[29] Murray RGE. The Higher Taxa, or, a Place for Everything...? In: Holt JG (ed). Bergey's Manual of Systematic Bacteriology, First Edition, Volume 1, The Williams and Wilkins Co, Baltimore 1984; p. 31-34.

[30] Garrity GM, Holt JG. The Road Map to the Manual. In: Garrity GM, Boone DR, Castenholz RW (eds), Bergey's Manual of Systematic Bacteriology, Second Edition, Volume 1, Springer, New York, 2001, p. 119-169.

[31] Ludwig W, Schleifer KH, Whitman WB. Class I. *Bacilli* class nov. *In*: De Vos P, Garrity GM, Jones D, N.R. Krieg W. Ludwig W, Rainey EA, Schleifer KH, Withman WB. Bergey's Manual of Systematic Bacteriology, second edition, vol 3 (The *Firmicutes*), Springer, Dordrecht, Heidelberg, London, New York **2009,** pp.19-20.

[32] List of new names and new combinations previously effectively, but not validly, published. List no. 132. Int J Syst Evol Microbiol 2010; 60:469-472 10.1099/ijs.0.022855-0 20458120

[33] Skerman VBD McGowan V Sneath PHA, eds. Approved Lists of Bacterial Names. Int J Syst Bacteriol 1980; 30:225-420 10.1099/00207713-30-1-225

[34] Prévot AR. *In*: Hauduroy P, Ehringer G, Guillot G, Magrou J, Prévot AR, Rosset. Urbain A (*eds*) Dictionnaire des Bactéries Pathogènes, 2nd ed., Masson, Paris, 1953, pp. 1-692.

[35] Fischer A Untersuchungen über bakterien. Jahrbücher für Wissenschaftliche Botanik 1985; 27:1-163

[36] Gibson T, Gordon RE. Genus I. *Bacillus* Cohn 1872; 174; Nom. gen. cons. Nomencl. Comm. Intern. Soc. Microbiol. 1937, 28; Opin. A. Jud. Comm. 1955, 39. In: Buchanan RE, Gibbons NE (eds), Bergey's Manual of Determinative Bacteriology, Eighth Edition, The Williams and Wilkins Co., Baltimore, 1974, p. 529-550.

[37] Ashburner M Ball CA Blake JA Botstein D Butler H Cherry JM Davis AP Dolinski K Dwight SS Eppig J T *et al* Gene ontology: tool for the unification of biology. The Gene Ontology Consortium. Nat Genet 2000; 25:25-29 10.1038/75556 10802651 PMC3037419

[38] Saitou N Nei M The neighbor-joining method: a new method for reconstructing phylogenetic trees. Mol Biol Evol 1987; 4:406-425 3447015 10.1093/oxfordjournals.molbev.a040454

[39] Tamura K Peterson D Peterson N Stecher G Nei M Kumar S MEGA5: molecular evolutionary genetics analysis using maximum likelihood, evolutionary distance, and maximum parsimony methods. Mol Biol Evol 2011; 28:2731-2739 10.1093/molbev/msr121 21546353 PMC3203626

[40] Kanso S Greene AC Patel BKC *Bacillus subterraneus* sp. nov., an iron- and manganese-reducing bacterium from a deep subsurface Australian thermal aquifer. Int J Syst Evol Microbiol 2002; 52:869-874 10.1099/ijs.0.01842-0 12054251

[41] Tiago I Pires C Mendes V Morais PV da Costa MS Verissimo A *Bacillus foraminis* sp. nov., isolated from a non-saline alkaline groundwater. Int J Syst Evol Microbiol 2006; 56:2571-2574 10.1099/ijs.0.64281-0 17082392

[42] Yoon JH Kang SS Lee KC Kho YH Choi SH Kang KH Park YH *Bacillus jeotgali* sp. nov., isolated from jeotgal, Korean traditional fermented seafood. Int J Syst Evol Microbiol 2001; 51:1087-1092 10.1099/00207713-51-3-1087 11411677

[43] Euzeby J Validation List no. 117. List of new names and new combinations previously effectively, but not validly, published. Int J Syst Evol Microbiol 2007; 57:1933-1934 10.1099/ijs.0.65495-0 17766848

[44] Perez-Ibarra BM Flores ME Garcia-Varela M Isolation and characterization of *Bacillus thioparus* sp. nov., chemolithoautotrophic, thiosulfate-oxidizing bacterium. FEMS Microbiol Lett 2007; 271:289-296 10.1111/j.1574-6968.2007.00729.x 17451444

[45] Ahmed I Yokota A Fujiwara T A novel highly boron tolerant bacterium, *Bacillus boroniphilus* sp. nov., isolated from soil, that requires boron for its growth. Extremophiles 2007; 11:217-224 10.1007/s00792-006-0027-0 17072687

[46] Heyrman J Vanparys B Logan NA Balcaen A Rodrıguez-Dıaz M Felske A De Vos P *Bacillus novalis* sp. nov., *Bacillus vireti* sp. nov., *Bacillus soli* sp. nov., *Bacillus bataviensis* sp. nov. and *Bacillus drentensis* sp. nov., from the Drentse A grasslands. Int J Syst Evol Microbiol 2004; 54:47-57 10.1099/ijs.0.02723-0 14742458

[47] Venkateswaran K Kempf M Chen F Satomi M Nicholson W Kern R *Bacillus nealsonii* sp. nov., isolated from a spacecraft-assembly facility, whose spores are gamma-radiation resistant. Int J Syst Evol Microbiol 2003; 53:165-172 10.1099/ijs.0.02311-0 12656168

[48] Lim JM Jeon CO Lee JR Park DJ Kim CJ *Bacillus kribbensis* sp. nov., isolated from a soil sample in Jeju, Korea. Int J Syst Evol Microbiol 2007; 57:2912-2916 10.1099/ijs.0.65227-0 18048748

[49] Seng P Rolain JM Fournier PE La Scola B Drancourt M Raoult D MALDI-TOF-mass spectrometry applications in clinical microbiology. Future Microbiol 2010; 5:1733-1754 10.2217/fmb.10.127 21133692

[50] Seng P Drancourt M Gouriet F La Scola B Fournier PE Rolain JM Raoult D Ongoing revolution in bacteriology: routine identification of bacteria by matrix-assisted laser desorption ionization time-of-flight mass spectrometry. Clin Infect Dis 2009; 49:543-551 10.1086/600885 19583519

[51] Field D Garrity G Gray T Morrison N Selengut J Sterk P Tatusova T Thomson N Allen MJ Angiuoli SV The minimum information about a genome sequence (MIGS) specification. Nat Biotechnol 2008; 26:541-547 10.1038/nbt1360 18464787 PMC2409278

[52] fastq-mcf program. http://code.google.com/p/ea-utils

[53] Prodigal. http://prodigal.ornl.gov

[54] GenBank database. http://www.ncbi.nlm.nih.gov/genbank

[55] RNAmmer 1.2 Server. http://www.cbs.dtu.dk/services/RNAmmer/

[56] Lagesen K Hallin P Rodland EA Staerfeldt HH Rognes T Ussery DW RNAmmer: consistent and rapid annotation of ribosomal RNA genes. Nucleic Acids Res 2007; 35:3100-3108 10.1093/nar/gkm160 17452365 PMC1888812

[57] Lowe TM Eddy SR tRNAscan-SE: a program for improved detection of transfer RNA genes in genomic sequence. Nucleic Acids Res 1997; 25:955-964 10.1093/nar/25.5.0955 9023104 PMC146525

[58] Kall L Krogh A Sonnhammer EL Advantages of combined transmembrane topology and signal peptide prediction--the Phobius web server. Nucleic Acids Res 2007; 35:W429-W432 10.1093/nar/gkm256 17483518 PMC1933244

[59] Rutherford K Parkhill J Crook J Horsnell T Rice P Rajandream MA Barrell B Artemis: sequence visualization and annotation. Bioinformatics 2000; 16:944-945 10.1093/bioinformatics/16.10.944 11120685

[60] Carver T Thomson N Bleasby A Berriman M Parkhill J DNAPlotter: circular and linear interactive genome visualization. Bioinformatics 2009; 25:119-120 10.1093/bioinformatics/btn578 18990721 PMC2612626

[61] Lechner M Findeiss S Steiner L Marz M Stadler PF Prohaska SJ Proteinortho: detection of (co-)orthologs in large-scale analysis. BMC Bioinformatics 2011; 12:124-133 10.1186/1471-2105-12-124 21526987 PMC3114741

